# A multi-institutional study of bladder-preserving therapy for stage II-IV bladder cancer: A Korean Radiation Oncology Group Study (KROG 14-16)

**DOI:** 10.1371/journal.pone.0209998

**Published:** 2019-01-17

**Authors:** Sang Jun Byun, Won Park, Kwan Ho Cho, Jaeho Cho, Ah Ram Chang, Ki Mun Kang, Jin Ho Kim, Jin Hee Kim

**Affiliations:** 1 Department of Radiation Oncology, Dongsan Medical Center, Keimyung University School of Medicine, Daegu, Republic of Korea; 2 Department of radiation oncology, Samsung Medical Center, Sungkyunkwan University School of Medicine, Seoul, Republic of Korea; 3 The Proton Therapy Center, Research Institute and Hospital, National Cancer Center, Goyang, Republic of Korea; 4 Department of Radiation Oncology, Severance Hospital, Yonsei University College of Medicine, Seoul, Republic of Korea; 5 Department of Radiation Oncology, Soonchunhyang University College of Medicine, Seoul, Republic of Korea; 6 Department of Radiation Oncology, Gyeongsang National University Hospital, Jinju, Republic of Korea; 7 Department of Radiation Oncology, Seoul National University College of Medicine, Seoul, Republic of Korea; Mayo Clinic in Arizona, UNITED STATES

## Abstract

**Background:**

Although radical cystectomy is a standard treatment in muscle-invasive bladder cancer, bladder preservation therapy including transurethral resection of bladder tumor, radiotherapy, and concurrent chemotherapy has been widely adopted, recently. This retrospective analysis was performed to evaluate the survival rates and prognostic factors related to treatment outcomes following bladder-preserving therapy including radiotherapy (RT) in bladder cancer with a curative intent.

**Materials and methods:**

We conducted a multi-institutional retrospective study of 152 patients with stage II-IV bladder cancer treated with curative RT between 2000 and 2010. There were 72 patients in stage II, 49 in stage III, and 31 in stage IV. Ninety-seven patients were treated with concurrent chemoradiotherapy and fifty-five with RT alone. Radiation was delivered to the pelvis (median 63 Gy), mainly with cisplatin. The median follow-up time was 35.5 months.

**Results:**

Sixty-nine patients (45.4%) showed a complete response to RT. The 5-year overall survival (OS) rate was 45.8%, the 5-year cause-specific survival (CSS) rate was 48.9%, and the 5-year disease-free survival (DFS) rate was 20.8%. Univariate analysis revealed significant differences in the following factors according to the survival rates: patient age, initial hemoglobin level, clinical T stage, clinical N stage, clinical stage group, tumor response to RT, hydronephrosis, and concurrent chemotherapy. Multivariate analysis also revealed a significant difference in patient age (p = 0.003 in OS, p<0.017 in CSS) and tumor response to RT (p = 0.002 in OS, p<0.001 in CSS). Concurrent chemotherapy was significantly different in the DFS rates (p = 0.046).

**Conclusions:**

The survival rates reported herein are comparable to those from other studies, and tumor response and concurrent chemoradiotherapy were significant prognostic factors for better survival rates. Further randomized studies are needed to elucidate the impact of RT in bladder cancer.

## Introduction

Bladder cancer is the 2^nd^ most frequent genitourinary tumor; the incidence rate is high worldwide, and the number of bladder cancer cases continues to increase in South Korea [1,2]. At the initial diagnosis, approximately 30% of bladder cancer cases exhibit muscle-invasive bladder cancer (MIBC) and, although the remaining cases are non-MIBC, 30% of patients with non-MIBC progress to invasive tumor during follow-up despite undergoing transurethral resection of bladder tumor (TURBT) [3].

Radical cystectomy (RC) with bilateral pelvic lymph node dissection (PLND) and urinary diversion has been a standard curative treatment for MIBC. Recent reports have revealed an approximately 50% 5-year local control rate and a 40–60% 5-year overall survival (OS) rate [4–6]. Additional reduction of the risk of recurrence, and improvement of OS have also been reported with the use of cisplatin-based chemotherapy [7]. However, RC-related morbidity and mortality cannot be disregarded. Although these results may have been influenced by several factors including patient age and medical co-morbidities, a recent study reported that the risk of perioperative mortality ranges from 0.8% to 2.7% [8,9].

For patients who are not eligible for radical surgery due to medical co-morbidities, and those who want to conserve their native bladder, definitive radiotherapy (RT) is an appropriate therapeutic option. Recently, use of TURBT and RT with or without concurrent chemotherapy has been adopted for bladder conservation, to improve patient quality of life without compromising oncologic outcomes [10]. Following bladder-preserving therapy, patients are expected to have survival outcomes that are comparable to previously reported RC results, as well as a better quality of life due to intact bladder function [6,11,12].

The authors retrospectively collected and analyzed clinical data from seven institutions affiliated with the Korean Radiation Oncology Group (KROG) and reviewed the current treatment status, analyzed clinical outcomes, and evaluated prognostic factors related to treatment outcomes following bladder-preserving therapy.

## Materials and methods

We retrospectively analyzed 152 patients with biopsy-proven, American Joint Committee on Cancer (AJCC, 7^th^ edition) stage II-IV bladder cancer, treated with bladder-preserving therapy from January 2000 to December 2010 at seven institutions participated in the KROG 14–16 study. This study was approved by the Institutional Review Board (IRB) of each university hospital or cancer center; Keimyung University Dongsan Medical Center IRB, Samsung Medical Center IRB, National Cancer Center IRB, Severance Hospital IRB, Soonchunhyang University Hospital Seoul IRB, Gyeongsang National University Hospital IRB, Seoul National University Hospital IRB, and the need for informed consent was waived.

Patients underwent physical examinations, cystoscopy with biopsy, complete blood count (CBC), liver function tests, urinalysis, chest radiography, and computed tomography (CT) or pelvic magnetic resonance imaging before treatment. Patient performance status was evaluated according to the guidelines of the Eastern Cooperative Oncology Group (ECOG). A CBC was performed at least once weekly during RT. When the absolute neutrophil count was <1,000/mm^3^ or the platelet count was < 50,000/mm^3^, treatment was interrupted or delayed until the patient’s condition recovered. Red blood cell transfusion was given to patients with hemoglobin levels less than 10.0 g/dL.

TURBT was performed to obtain an accurate diagnosis and for maximal tumor removal. If needed, repeated TURBT was performed during the follow-up period in patients with suspected recurrence. Most patients underwent TURBT under spinal anesthesia. Usually, external beam RT was delivered using 6, 10, 15, or 20 mega-voltage photon beams with a four-field box technique, for 5 days each week over 7 weeks. The median fraction size was 1.8 Gy (range: 1.8–2.5). Irradiated fields were defined according to common criteria in the most patients, but they were sometimes changed due to each physician's decision. For the initial pelvic fields, in most patients, the superior border was at the middle of the sacroiliac joint or at the L5–S1 interspace, and the interior border was at or just below the bottom of the obturator foramen. The inferior border was sometimes extended to the bottom of the ischial tuberosity, depending on disease involvement, with the prostatic urethra or bladder neck. The lateral border was 1.5–2.0 cm lateral to the true pelvis, to encompass the bladder and the pelvic lymph nodes. On the lateral portal, the anterior border was placed in front of the bladder and the posterior border was set with an at least 3-cm margin behind the posterior border bladder wall. The portals were reduced for most patients after 45 Gy (1.8 Gy per fraction), and a follow-up CT study was performed. Boost treatment included an initial gross tumor volume with a 2-cm margin. Neoadjuvant chemotherapy adapted before TURBT and RT, and concurrent chemotherapy with RT were administered by intravenous infusion. Cisplatin was mainly administered as a concurrent chemotherapeutic agent, and 40 mg/m^2^ was given every week on the first day of the chemotherapy cycle (D1, D8, D15, and D22) within 16 hours after RT. Another regimen (cisplatin and paclitaxel) was also adapted, and 50 mg/m^2^ of paclitaxel (D1, D8, and D15) and 15 mg/m^2^ of cisplatin (D1–3, D8–10, D15–17) were combined.

After completion of treatment, all patients were evaluated by radiation oncologists and urologic oncologists at 3-month intervals for 1 year and at least 6 months thereafter. Complete response (CR) was defined as no evidence of a gross tumor upon follow-up cystoscopic examination and biopsy, pelvic CT or MRI at 3 months after completion of treatment. Partial response (PR) was defined as T < 1 according to the Radiation Therapy Oncology Group. Loco-regional failure was defined as any disease lesions within pelvic radiation fields that developed 3 months later and thereafter. Distant metastasis was defined as any disease occurring outside of the pelvic radiation fields.

The OS rate was calculated from the time between diagnosis and date of death due to cause any death as caused from cancer. Cause-specific survival (CSS) was calculated from the time between diagnosis and date of death related to bladder cancer. Disease-free survival (DFS) was calculated from the end of primary treatment to the date of diagnosing loco-regional recurrence or distant metastasis in patients with CR after RT. Kaplan-Meier analysis was performed to estimate the OS, CSS, and DFS rates, with the log-rank test used to assess prognostic significance and the Cox proportional hazards model used for multivariate analysis. A chi-square test was used to analyze the relationship between factors among the two groups. A p-value less than 0.05 was considered statistically significant. Statistical analyses were performed using SPSS software (ver. 21.0; IBM Corp., Armonk, NY, USA).

## Results

The pretreatment characteristics of the 152 patients are listed in S1 Table. Patient age ranged from 37 to 94 years with a median age of 72 years. In total, 121 (79.6%) patients were male and 31 (20.4%) were female. Gross hematuria was the most common initial symptom in 125 patients (82.2%), and 13 patients (8.6%) suffered from dysuria. Patients had several underlying diseases, such as angina pectoris or acute myocardial infarction (10 patients), cerebrovascular disease (6 patients), hypertension (46 patients), and diabetes mellitus (33 patients). Forty-eight patients (31.6%) had a history of cigarette smoking.

In total, 141 (92.8%) patients showed a good performance status (ECOG grade of 0 or 1). With the exception of 10 cases, all patients underwent TURBT. Of the 142 patients who underwent TURBT, 92 (64.7%) underwent TURBT once and the other 50 (35.3%) underwent TURBT more than once. Complete TURBT was observed in 99 patients (65.1%). Urothelial cell carcinoma was the dominant type upon pathological examination (96.7%). Approximately 80% of the patients had high-grade tumors. Half of all patients were in clinical stage T2, and more than 80% were had a clinically negative lymph node status. The AJCC stage was II in 72 patients (47.4%), III in 49 patients (32.2%), and IV in 31 patients (20.4%). Ninety-seven patients (63.8%) had a solitary tumor in the bladder, and forty-six (30.3%) had multiple tumors. Hydronephrosis was reported in 40 patients (26.3%), and 111 patients (73.0%) had normal kidneys. The initial hemoglobin level ranged from 6.8 to 16.5 g/dL, with a median level of 12.1 g/dL.

Neoadjuvant chemotherapy prior to TURBT and RT was administered in 50 patients (32.9%). Concurrent chemotherapy with RT was adopted in 97 patients (63.8%), and the chemotherapeutic regimen mainly comprised cisplatin-based agents. The total radiation dose ranged from 43.2 to 70.2 Gy (median 63 Gy, typically 1.8 Gy per fraction). The median dose administered to the pelvic lymph nodal area was 45 Gy and that to the bladder tumor was 63 Gy. More details of treatment variables are summarized in the S2 Table.

Three months after completion of treatment, 69 patients (45.4%) showed CR and 46 (30.3%) showed PR (S3 Table). Disease had progressed in 15 patients (9.9%) and 14 (9.2%) had a stable disease status. Of the 69 patients with CR, 38 patients (55.1%) were alive without evidence of disease on last follow-up. Of the rest 31 patients with failure after CR, loco-regional failure was reported in 25 patients. The most common site of loco-regional failure after complete response was the bladder (24 patients), and pelvic nodal failure was in 1 patient only. For entire cohort, distant failure was reported in 40 patients (26.3%). The sites of distant metastasis were various, and included bone, lung, brain, and lymph nodes above the common iliac chain. S4 Table also showed clinical outcomes according to T stage. Higher rates of lymph nodal metastasis and presence of gross residual tumor after TURBT were related to advanced T stage. Although there was no statistical significance in tumor response after RT, 5-year OS and CSS were significantly better in lower T stage group.

The 5-year OS rate was 45.8% (Panel A of S1 Fig), the 5-year CSS rate was 48.9% (Panel B of S1 Fig), and the 5-year DFS rate was 20.8% (Panel C of S1 Fig) in all patients. The 3-year OS rate was 56.9%, the 5-year CSS rate was 59.1%, and the 5-year DFS rate was 25.6%. Univariate analysis revealed significant differences in patient age, clinical N stage, clinical stage group, and tumor response to RT (S2 Fig) according to the OS, CSS, and DFS rates. The initial hemoglobin level, clinical T stage, and hydronephrosis status were statistically significant in the OS and CSS rates, and concurrent chemotherapy with RT (S3 Fig) was also significant when analyzing the DFS rates (S5 Table). There was no statistical significance between neoadjuvant chemotherapy and survival outcomes including OS, CSS, and DFS. Multivariate analysis revealed a significant difference in patient age (OS: 95% confidence interval [CI], 0.32 to 0.80; hazard ratio [HR], 0.51; p = 0.003; CSS: 95% CI, 0.35 to 0.90; HR, 0.56; p<0.017) and tumor response to RT (OS: 95% CI, 0.30 to 0.76; HR, 0.48; p = 0.002; CSS: 95% CI, 0.27 to 0.69; HR, 0.43; p<0.001) according to the OS and CSS rates. Concurrent chemotherapy (95% CI, 0.44 to 0.99; HR, 0.66; p = 0.046) was significantly different according to the DFS rate (S6 Table).

## Discussion

Although RC remains the standard therapy for treatment of MIBC, several recent reports have shown comparable clinical outcomes using bladder-preserving therapy [13]. The present work was a nationwide, multi-institutional retrospective study. The authors analyzed clinical data to estimate the survival rates for MIBC, and to identify several prognostic factors related to survival rates and failure patterns following bladder-preserving therapy in MIBC; however, there were differences in treatment protocols, including use of fractionation schemes in RT, chemotherapeutic regimens, and cycles among institutions.

The tumor response rates after RT and survival rates reported herein may appear unsatisfactory compared to previous reports. The overall CR rate following bladder-preserving therapy, including tri-modality therapy, which in turn includes TURBT, concurrent chemotherapy, and RT, was approximately 73%, ranging from 54.7% to 93% [13]. However, more than one-third of the patients analyzed herein were not administered concurrent chemotherapy due to their severe medical co-morbidities; therefore, the comparison was not direct. Of the 97 patients who received concurrent chemotherapy and RT, 48 patients (49.5%) showed CR after tri-modality therapy, although the chemotherapeutic regimens varied. Among previous reports, the 5-year OS rate was approximately 50%, ranging from 36% to 74%, and the 5-year CSS rate ranged from 50% to 82% [14–18]. A recent report of long-term outcomes by the Radiation Therapy Oncology Group (RTOG) showed a 5-year OS rate of 57% and a 5-year CSS rate of 71% [12]. Regarding the characteristics of the patient in the RTOG study, approximately two-thirds of patients were less than 70 years old and 60.6% were in clinical stage T2. Only 10.6% of the patients had hydronephrosis. Considering that several different clinical parameters are associated with a favorable outcome, such as unifocal tumor, absence of hydronephrosis, clinical stage T2, and smaller tumor size [19,20], the patient characteristics described in the present study were less favorable: half of all the patients were in clinical stage T3 or T4, 30.3% had multiple tumors, 80.2% had a high-grade tumor, and 26.3% with hydronephrosis. In the report by US ha et al., they reported laparoscopic versus open radical cystectomy results in South Korea as a single institutional retrospective study [21]. Three-year OS rates were 64.2% - 72.6% and 3-year CSS rates were 73.0% - 75.3%, but direct comparison to our results seems to be difficult due to the differences in patient characteristics including T stage, N stage, and patient’s medical conditions.

In contrast to RC, treatment failure in the bladder is a common concern in bladder-preserving therapy, particularly for MIBC in the preserved bladder [22]. In the present study, 24 patients (34.7%) with CR showed in-bladder recurrence. Among previous studies, in-bladder recurrence rates ranged from 19% to 58%, and MIBC recurrence was approximately half as prevalent as intra-vesical recurrence [20,23,24]. Rodel et al. [20] reported 5-year cumulative bladder cancer recurrence and MIBC recurrence rates of 41% and 28%, respectively. Regarding in-bladder recurrence sites, Tunio et al. [16] reported that 21% of patients with initial CR after tri-modality therapy showed MIBC recurrence, of which 69% were within the original MIBC site.

There are several methods for achieving better bladder control: radiation dose escalation is one possible solution. Hafeez et al. [25] reported the feasibility of simultaneous integrated boost to 70 Gy without MIBC recurrence, even with low rates of late toxicity. Murthy et al. [26] also reported the use of dose escalation during image-guided intensity-modulated RT in a prospective study. However, it is required to define accurate tumor location for dose escalation. Recently, with using image-guide techniques such as cone-beam CT, lipiodol injection around the residual tumor or tumor bed was introduced [27,28]. The procedure consists of intramuscular injection of lipiodol, and verification of lipiodol location on cone-beam CT during daily RT. However, wide use of this technique would be premature due to a lack of sufficient clinical data.

As mentioned, cisplatin-based concurrent chemotherapy and radiotherapy has been adapted for MIBC patients who are unfit for surgery. Recently, advances in cancer treatment also have emphasized the role of the immune system in tumor growth and progression, and intra-tumoral CD8+ T cells have been reported to associated with prognosis in cancer patients [29,30]. Two immune checkpoint inhibitors including Atezoliumab, Pembrolizumab were approved by US Food and Drug Administration (FDA) and European Medicines Agency (EMA) as first-line therapy for cisplatin-ineligible advanced urothelial cancer in 2017 [31]. For both MIBC and non-MIBC, there are also many ongoing clinical trials evaluating immune checkpoint inhibitors as a monotherapy and combination therapy, and together with the identification of biomarkers, it would be elucidating the antitumor activity and helpful to improve the efficacy of the treatments.

To define appropriate irradiation field is another concern related to loco-regional failure in RT. In BC2001 trial, although clinical lymph node negative patients were included and the irradiated target was bladder, pelvic lymph node failures were reported in only 4.9% after concurrent chemoradiotherapy [14]. In the present study, pelvic lymph node failure was also seen in 1.2% (1 patient) among the 85 patients after concurrent chemoradiotherapy without clinical lymph node involvement. Indeed, a randomized study comparing bladder only irradiation (65 Gy) with pelvic lymph nodal irradiation (45 Gy to whole pelvis and 20 Gy bladder boost) showed similar rates in pelvic nodal failure [16]. These results imply that RT fields including bladder only could be feasible in selected patients and concurrent chemotherapy might also have targeted microscopic lymph nodal disease.

Our results identified several prognostic factors related to survival outcome, including patient age, tumor response to RT and concurrent chemotherapy. The combination of concurrent chemotherapy and RT has been reported to show better clinical outcomes, including OS and DFS [14,32]. In the Bladder Cancer 2001 (BC2001) study, the authors demonstrated improved loco-regional DFS (67% vs. 54%, p = 0.03) and a trend toward increased OS (48% vs. 35%, p = 0.16), although the chemotherapeutic regimen was a combination of 5-fluorouracil and mitomycin C [14]. Currently, weekly cisplatin-based chemotherapy with RT is the most common combination treatment for MIBC, and it has been also administered, even as a neoadjuvant or adjuvant chemotherapy (eg, MVAC and gemcitabine/cisplatin) with the intention of eradicating distant micro-metastases that are not treated by RT [4].

In the present study, there are several limitations, and foremost the limitations were originated from retrospective design. Therefore, this study is limited by several biases including patient selection, data acquisition. It should be also noticed that patient data was heterogenous from seven distinct institutions, and the patients were treated by multiple urologists, radiation oncologists, and medical oncologists. Although our nationwide study represents the outcomes in South Korea, this may also limit generalize the results to other regions.

## Conclusion

Clinical outcomes including tumor response and survival rates are comparable to those from other studies, and tumor response and concurrent chemotherapy are significant prognostic factors for better survival rates. Bladder preserving therapy seems to be an effective treatment accomplishing competent oncologic outcomes with retaining native bladder, however careful regular follow-up after treatment is also necessary to prevent and detect subsequent bladder recurrence. Further randomized studies are needed to elucidate the impact of RT in bladder cancer.

## Supporting information

S1 Fig **Kaplan-Meier estimates of overall survival (OS, A), cause-specific survival (CSS, B), and disease-free survival (DFS, C).** Number at risk for OS, CSS, and DFS was indicated, respectively.
(TIF)

S2 Fig Kaplan-Meier estimates of cause-specific survival (CSS) according to tumor response to radiotherapy. (TIF)

S3 Fig Kaplan-Meier estimates of disease-free survival (DFS) according to concurrent chemoradiotherapy. (TIF)

S1 Table Patient characteristics. (DOCX)

S2 Table Treatment variables. (DOCX)

S3 Table Tumor response and failure patterns. (DOCX)

S4 Table Clinical outcomes according to clinical T stage. (DOCX)

S5 Table Univariate analysis of prognostic factors to OS, CSS and DFS. (DOCX)

S6 Table Multivariate analysis of prognostic factors of OS, CSS, and DFS. (DOCX)

S1 File KROG 14–16 data.xlsx. (XLSX)

## References

[1] Chavan S, Bray F, Lortet-Tieulent J, Goodman M, Jemal A. International variations in bladder cancer incidence and mortality. Eur Urol. 2014; 66(1):59–73. 10.1016/j.eururo.2013.10.001 24451595

[2] Song W, Jeon HG. Incidence of kidney, bladder, and prostate cancers in Korea: An update. Korean J Urol. 2015; 56(6):422–8. 10.4111/kju.2015.56.6.422 26078838 PMC4462631

[3] Stenzl A, Cowan NC, De Santis M, Kuczyk MA, Merseburger AS, Ribal MJ, et al Treatment of muscle-invasive and metastatic bladder cancer: update of the EAU guidelines. Eur Urol. 2011; 59(6):1009–18. 10.1016/j.eururo.2011.03.023 21454009

[4] Efstathiou JA, Spiegel DY, Shipley WU, Heney NM, Kaufman DS, Niemierko A, et al Long-term outcomes of selective bladder preservation by combined-modality therapy for invasive bladder cancer: the MGH experience. Eur Urol. 2012; 61(4):705–11. 10.1016/j.eururo.2011.11.010 22101114

[5] Grossman HB, Natale RB, Tangen CM, Speights VO, Vogelzang NJ, Trump DL, et al Neoadjuvant chemotherapy plus cystectomy compared with cystectomy alone for locally advanced bladder cancer. N Engl J Med. 2003; 349(9):859–66. 10.1056/NEJMoa022148 12944571

[6] Stein JP, Lieskovsky G, Cote R, Groshen S, Feng AC, Boyd S, et al Radical cystectomy in the treatment of invasive bladder cancer: long-term results in 1,054 patients. J Clin Oncol. 2001; 19(3):666–75. 10.1200/JCO.2001.19.3.666 11157016

[7] Advanced Bladder Cancer Meta-analysis C. Neoadjuvant chemotherapy in invasive bladder cancer: a systematic review and meta-analysis. Lancet. 2003; 361(9373):1927–34. 12801735 10.1016/s0140-6736(03)13580-5

[8] Novotny V, Hakenberg OW, Wiessner D, Heberling U, Litz RJ, Oehlschlaeger S, et al Perioperative complications of radical cystectomy in a contemporary series. Eur Urol. 2007; 51(2):397–401; discussion 01–2. 10.1016/j.eururo.2006.06.014 16905242

[9] Konety BR, Allareddy V, Herr H. Complications after radical cystectomy: analysis of population-based data. Urology. 2006; 68(1):58–64. 10.1016/j.urology.2006.01.051 16806414

[10] McHaffie DR, Kruser TJ, Gaston K, Mahoney J, Graham D, Haake M. Chemoradiation for organ preservation in the treatment of muscle-invasive bladder cancer. Urol Oncol. 2016; 34(6):271–8. 10.1016/j.urolonc.2016.03.011 27108225

[11] Shariat SF, Karakiewicz PI, Palapattu GS, Lotan Y, Rogers CG, Amiel GE, et al Outcomes of radical cystectomy for transitional cell carcinoma of the bladder: a contemporary series from the Bladder Cancer Research Consortium. J Urol. 2006; 176(6 Pt 1):2414–22; discussion 22. 10.1016/j.juro.2006.08.004 17085118

[12] Mak RH, Hunt D, Shipley WU, Efstathiou JA, Tester WJ, Hagan MP, et al Long-term outcomes in patients with muscle-invasive bladder cancer after selective bladder-preserving combined-modality therapy: a pooled analysis of Radiation Therapy Oncology Group protocols 8802, 8903, 9506, 9706, 9906, and 0233. J Clin Oncol. 2014; 32(34):3801–9. 10.1200/JCO.2014.57.5548 25366678 PMC4239302

[13] Ploussard G, Daneshmand S, Efstathiou JA, Herr HW, James ND, Rodel CM, et al Critical analysis of bladder sparing with trimodal therapy in muscle-invasive bladder cancer: a systematic review. Eur Urol. 2014; 66(1):120–37. 10.1016/j.eururo.2014.02.038 24613684

[14] James ND, Hussain SA, Hall E, Jenkins P, Tremlett J, Rawlings C, et al Radiotherapy with or without chemotherapy in muscle-invasive bladder cancer. N Engl J Med. 2012; 366(16):1477–88. 10.1056/NEJMoa1106106 22512481

[15] Choudhury A, Swindell R, Logue JP, Elliott PA, Livsey JE, Wise M, et al Phase II study of conformal hypofractionated radiotherapy with concurrent gemcitabine in muscle-invasive bladder cancer. J Clin Oncol. 2011; 29(6):733–8. 10.1200/JCO.2010.31.5721 21205754

[16] Tunio MA, Hashmi A, Qayyum A, Mohsin R, Zaeem A. Whole-pelvis or bladder-only chemoradiation for lymph node-negative invasive bladder cancer: single-institution experience. Int J Radiat Oncol Biol Phys. 2012; 82(3):e457–62. 10.1016/j.ijrobp.2011.05.051 21945107

[17] Lagrange JL, Bascoul-Mollevi C, Geoffrois L, Beckendorf V, Ferrero JM, Joly F, et al Quality of life assessment after concurrent chemoradiation for invasive bladder cancer: results of a multicenter prospective study (GETUG 97–015). Int J Radiat Oncol Biol Phys. 2011; 79(1):172–8. 10.1016/j.ijrobp.2009.10.038 20385453

[18] Zapatero A, Martin De Vidales C, Arellano R, Ibanez Y, Bocardo G, Perez M, et al Long-term results of two prospective bladder-sparing trimodality approaches for invasive bladder cancer: neoadjuvant chemotherapy and concurrent radio-chemotherapy. Urology. 2012; 80(5):1056–62. 10.1016/j.urology.2012.07.045 22999456

[19] Koga F, Yoshida S, Kawakami S, Kageyama Y, Yokoyama M, Saito K, et al Low-dose chemoradiotherapy followed by partial or radical cystectomy against muscle-invasive bladder cancer: an intent-to-treat survival analysis. Urology. 2008; 72(2):384–8. 10.1016/j.urology.2008.03.017 18455771

[20] Rodel C, Grabenbauer GG, Kuhn R, Papadopoulos T, Dunst J, Meyer M, et al Combined-modality treatment and selective organ preservation in invasive bladder cancer: long-term results. J Clin Oncol. 2002; 20(14):3061–71. 10.1200/JCO.2002.11.027 12118019

[21] Ha US, Kim SI, Kim SJ, Cho HJ, Hong SH, Lee JY, et al Laparoscopic versus open radical cystectomy for the management of bladder cancer: mid-term oncological outcome. Int J Urol. 2010; 17(1):55–61. 10.1111/j.1442-2042.2009.02425.x 19930499

[22] Koga F, Kihara K. Selective bladder preservation with curative intent for muscle-invasive bladder cancer: a contemporary review. Int J Urol. 2012; 19(5):388–401. 10.1111/j.1442-2042.2012.02974.x 22409269

[23] Hagan MP, Winter KA, Kaufman DS, Wajsman Z, Zietman AL, Heney NM, et al RTOG 97–06: initial report of a phase I-II trial of selective bladder conservation using TURBT, twice-daily accelerated irradiation sensitized with cisplatin, and adjuvant MCV combination chemotherapy. Int J Radiat Oncol Biol Phys. 2003; 57(3):665–72. 14529770 10.1016/s0360-3016(03)00718-1

[24] Rivera I, Wajsman Z. Bladder-sparing treatment of invasive bladder cancer. Cancer Control. 2000; 7(4):340–6. 10.1177/107327480000700403 10895128

[25] Hafeez S, Warren-Oseni K, McNair HA, Hansen VN, Jones K, Tan M, et al Prospective Study Delivering Simultaneous Integrated High-dose Tumor Boost (</ = 70 Gy) With Image Guided Adaptive Radiation Therapy for Radical Treatment of Localized Muscle-Invasive Bladder Cancer. Int J Radiat Oncol Biol Phys. 2016; 94(5):1022–30. 10.1016/j.ijrobp.2015.12.379 27026308

[26] Murthy V, Masodkar R, Kalyani N, Mahantshetty U, Bakshi G, Prakash G, et al Clinical Outcomes With Dose-Escalated Adaptive Radiation Therapy for Urinary Bladder Cancer: A Prospective Study. Int J Radiat Oncol Biol Phys. 2016; 94(1):60–6. 10.1016/j.ijrobp.2015.09.010 26547385

[27] Baumgarten AS, Emtage JB, Wilder RB, Biagioli MC, Gupta S, Spiess PE. Intravesical lipiodol injection technique for image-guided radiation therapy for bladder cancer. Urology. 2014; 83(4):946–50. 10.1016/j.urology.2013.09.058 24397940

[28] Chai X, van Herk M, van de Kamer JB, Remeijer P, Bex A, Betgen A, et al Behavior of lipiodol markers during image guided radiotherapy of bladder cancer. Int J Radiat Oncol Biol Phys. 2010; 77(1):309–14. 10.1016/j.ijrobp.2009.08.019 20137863

[29] Schreiber RD, Old LJ, Smyth MJ. Cancer immunoediting: integrating immunity's roles in cancer suppression and promotion. Science (New York, NY). 2011; 331(6024):1565–70. 10.1126/science.1203486 21436444

[30] Fridman WH, Zitvogel L, Sautes-Fridman C, Kroemer G. The immune contexture in cancer prognosis and treatment. Nature reviews Clinical oncology. 2017; 14(12):717–34. 10.1038/nrclinonc.2017.101 28741618

[31] Rouanne M, Roumiguie M, Houede N, Masson-Lecomte A, Colin P, Pignot G, et al Development of immunotherapy in bladder cancer: present and future on targeting PD(L)1 and CTLA-4 pathways. World journal of urology. 2018 10.1007/s00345-018-2332-5 29855698

[32] Byun SJ, Kim JH, Oh YK, Kim BH. Concurrent chemoradiotherapy improves survival outcome in muscle-invasive bladder cancer. Radiat Oncol J. 2015; 33(4):294–300. 10.3857/roj.2015.33.4.294 26756029 PMC4707212

