# Supplementary figures and images for: A multi-institutional study of bladder-preserving therapy for stage II-IV bladder cancer: A Korean Radiation Oncology Group Study (KROG 14-16)
Source: PLoS One. 2019 Jan 17;14(1):e0209998. doi: 10.1371/journal.pone.0209998 (PMC6336268; doi:10.1371/journal.pone.0209998)

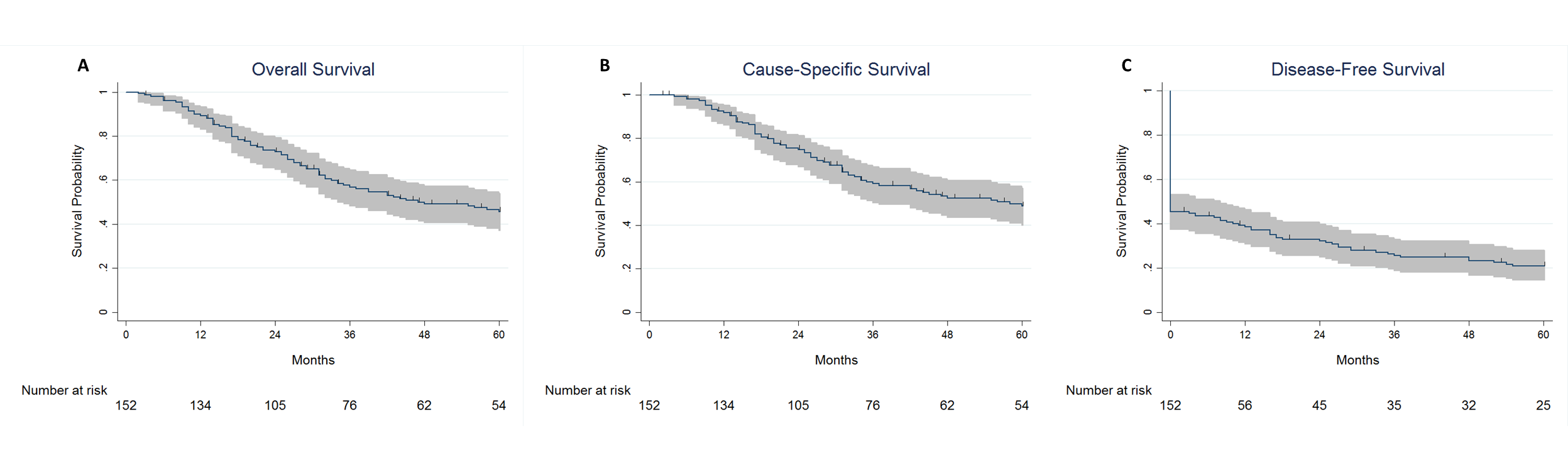

Supplement: S1 Fig — Kaplan-Meier estimates of overall survival (OS, A), cause-specific survival (CSS, B), and disease-free survival (DFS, C). Number at risk for OS, CSS, and DFS was indicated, respectively. (TIF) [file pone.0209998.s001.tif]

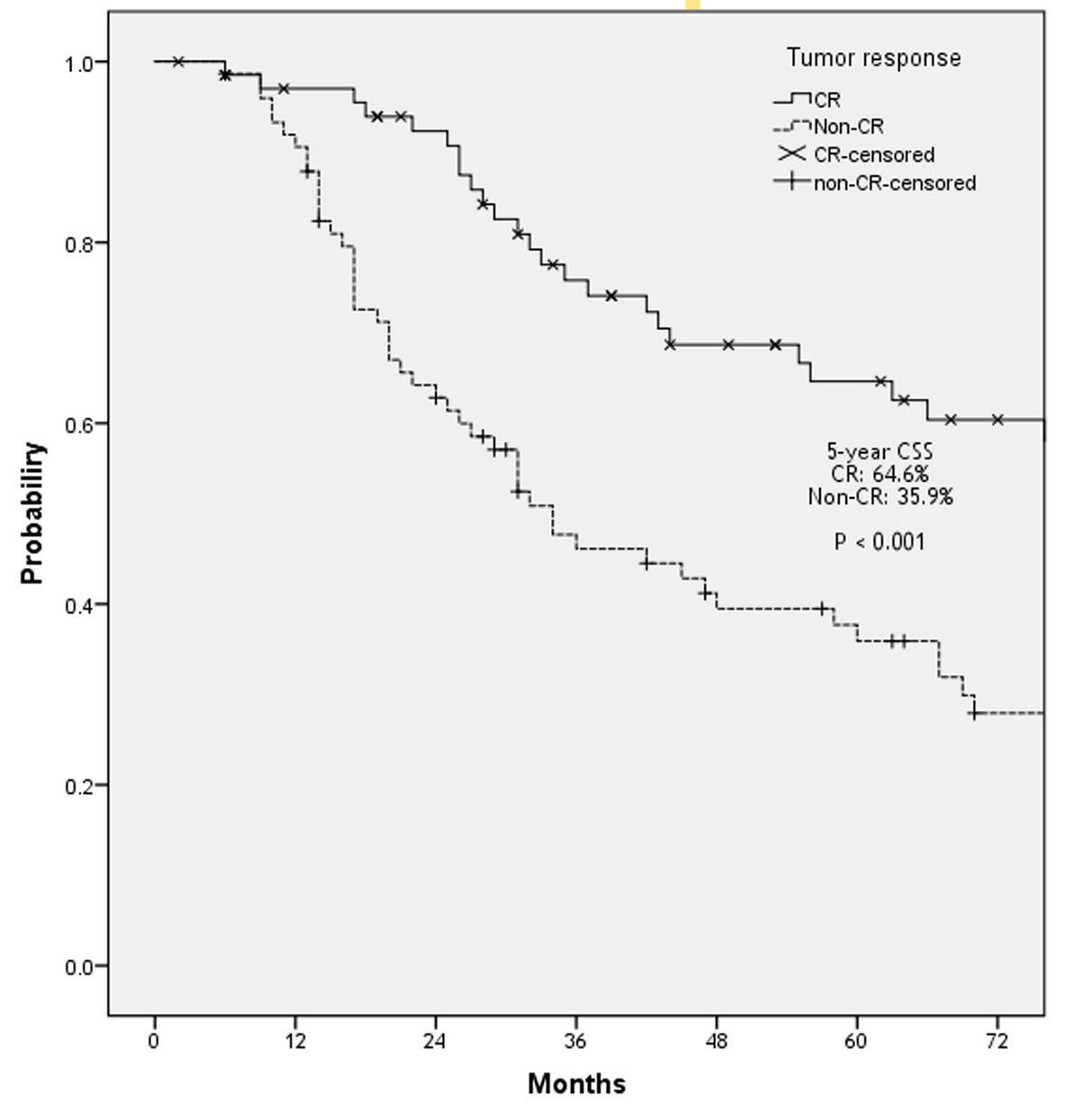

Supplement: S2 Fig — (TIF) [file pone.0209998.s002.tif]

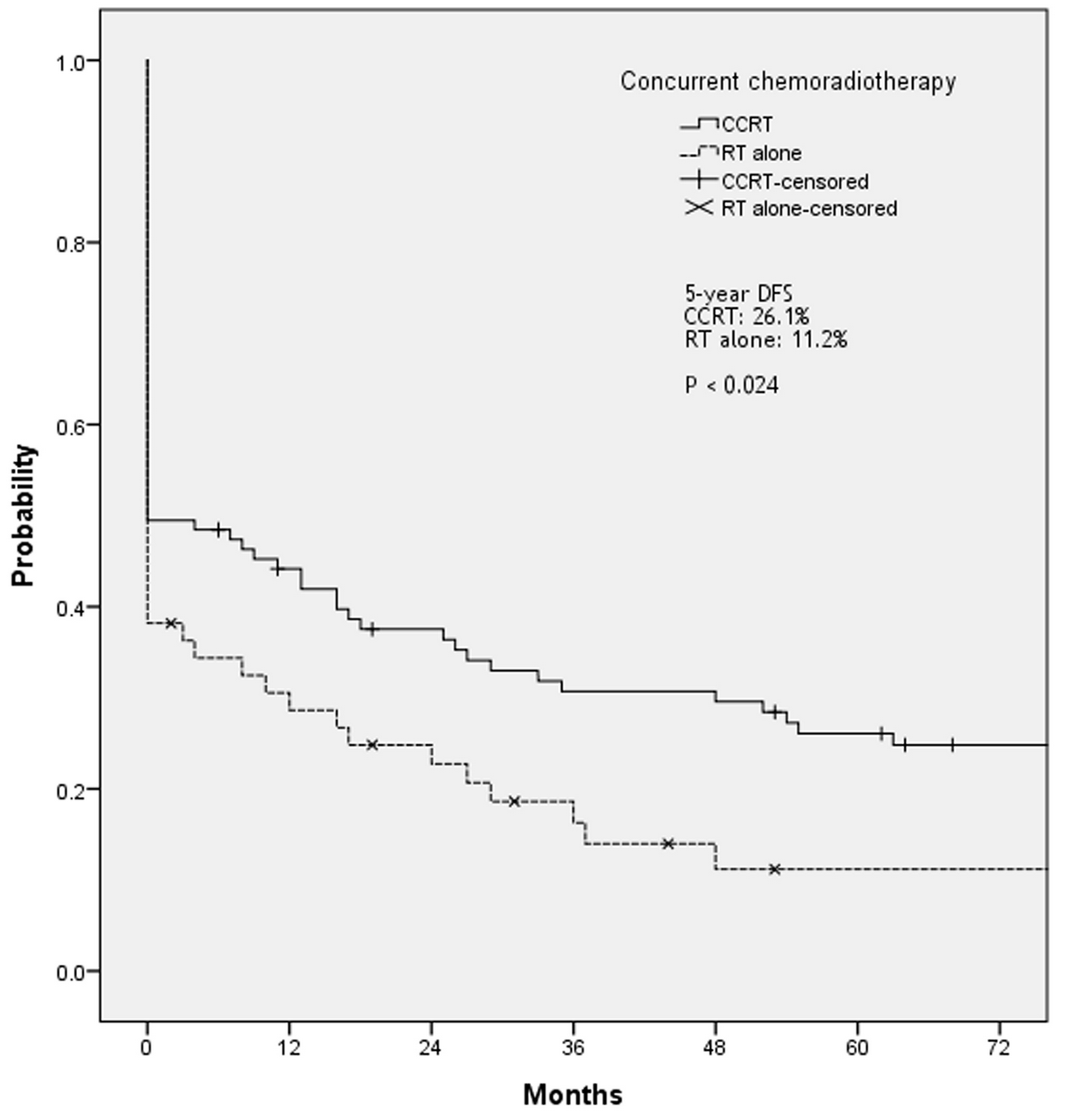

Supplement: S3 Fig — (TIF) [file pone.0209998.s003.tif]
