# Supplementary material for: A multi-institutional study of bladder-preserving therapy for stage II-IV bladder cancer: A Korean Radiation Oncology Group Study (KROG 14-16)
Source: PLoS One. 2019 Jan 17;14(1):e0209998. doi: 10.1371/journal.pone.0209998 (PMC6336268; doi:10.1371/journal.pone.0209998)
Supplement: S1 Table — (DOCX) [file pone.0209998.s004.docx]

**S1 Table. Patient characteristics.**

| **Variables** | **No.** |  | **Variables** | **No.** |
| --- | --- | --- | --- | --- |
| **Age (year)** |  |  | **Tumor grade** |  |
| Median (range) | 72 (37 – 94) |  | High | 122 (80.2%) |
| **Sex** |  |  | Low | 18 (11.8%) |
| Male | 121 (79.6%) |  | Unknown | 12 (7.9%) |
| Female | 31 (20.4%) |  | **Multiplicity** |  |
| **ECOG** |  |  | Solitary | 97 (63.8%) |
| 0 | 56 (36.8%) |  | Multiple | 46 (30.3%) |
| 1 | 85 (55.9%) |  | Diffuse | 4 (2.6%) |
| 2 | 11 (7.2%) |  | Unknown | 5 (3.3%) |
| **Smoking history** |  |  | **Clinical T stage** |  |
| Yes | 48 (31.6%) |  | 2 | 75 (49.3%) |
| No | 104 (68.4%) |  | 3 | 54 (35.5%) |
| **Hypertension** |  |  | 4 | 23 (15.1%) |
| Yes | 46 (30.3%) |  | **Clinical N stage** |  |
| No | 106 (69.7%) |  | 0 | 125 (82.2%) |
| **Diabetes** |  |  | 1 | 19 (12.5%) |
| Yes | 33 (21.7%) |  | 2 | 6 (3.9%) |
| No | 119 (78.3%) |  | 3 | 2 (1.3%) |
| **Initial symptom** |  |  | **Clinical stage group** |  |
| Hematuria | 125 (82.2%) |  | II | 72 (47.4%) |
| Dysuria | 13 (8.6%) |  | III | 49 (32.2%) |
| Others | 14 (9.2%) |  | IV | 31 (20.4%) |
| **Initial hemoglobin (g/dL)** |  |  | **Hydronephrosis** |  |
| Median (range) | 12.1 (6.8 – 16.5) |  | Present | 40 (26.3%) |
| **Pathology** |  |  | Absent | 111 (73.0%) |
| Urothelial carcinoma | 147 (96.7%) |  | Unknown | 1 (0.7%) |
| Others | 5 (3.3%) |  |  |  |
|  |  |  |  |  |
