# Supplementary material for: A multi-institutional study of bladder-preserving therapy for stage II-IV bladder cancer: A Korean Radiation Oncology Group Study (KROG 14-16)
Source: PLoS One. 2019 Jan 17;14(1):e0209998. doi: 10.1371/journal.pone.0209998 (PMC6336268; doi:10.1371/journal.pone.0209998)
Supplement: S2 Table — (DOCX) [file pone.0209998.s005.docx]

**S2 Table. Treatment variables.**

| **Variables** | **No. (%)** | **Variables** | **No. (%)** |
| --- | --- | --- | --- |
| **TURBT** |  | **Radiotherapy** |  |
| Yes | 142 (93.4) | Total dose | 43.2 – 70.2 Gy |
| No | 10 (6.6) |  | (median, 63) |
| **No. of TURBT** |  | Dose to tumor | 43.2 – 70.2 Gy |
| 1 | 92 (64.7) |  | (median, 63) |
| 2 | 26 (18.3) | Dose to lymph nodes | 39.6 – 66 Gy |
| More than 3 | 24 (16.9) |  | (median, 45) |
| **NACT** |  | **Concurrent chemotherapeutic regimen** | |
| Yes | 50 (32.9) | Cisplatin | 87 (89.7) |
| No | 102 (67.1) | Cisplatin + paclitaxel | 3 (3.1) |
| **Concurrent chemotherapy** |  | Others | 7 (7.2) |
| Yes | 97 (63.8) | **Concurrent chemotherapeutic cycle(s)** | |
| No | 55 (36.2) | 1 - 3 | 82 (84.5) |
|  |  | More than 4 | 15 (15.5) |

Abbreviations: TURBT = transurethral resection of bladder tumor; NACT = neoadjuvant chemotherapy.
