# Supplementary material for: A multi-institutional study of bladder-preserving therapy for stage II-IV bladder cancer: A Korean Radiation Oncology Group Study (KROG 14-16)
Source: PLoS One. 2019 Jan 17;14(1):e0209998. doi: 10.1371/journal.pone.0209998 (PMC6336268; doi:10.1371/journal.pone.0209998)
Supplement: S3 Table — (DOCX) [file pone.0209998.s006.docx]

**S3 Table. Tumor response and failure patterns.**

| **Variables** | **No. (%)** | **Variables** | **No. (%)** |
| --- | --- | --- | --- |
| **Tumor response after radiotherapy** |  | **Distant failure** |  |
| Complete response | 69 (45.4) | Yes | 40 (26.3) |
| Partial response | 46 (30.3) | No | 112 (73.7) |
| Progression | 15 (9.9) | **Status** |  |
| Stable disease | 14 (9.2) | No evidence of disease | 38 (25) |
| Unknown | 8 (5.3) | Alive with disease | 19 (12.5) |
| **Failure pattern after complete response** |  | Intercurrent death | 10 (6.6) |
| No evidence of disease | 38 (55.1) | Died of disease | 60 (39.5) |
| Bladder recurrence only | 17 (24.6) | Unknown | 25 (16.4) |
| Bladder recurrence and distant failure | 7 (10.1) |  |  |
| Distant failure only | 6 (8.7) |  |  |
| Pelvic lymph node recurrence only | 1 (1.5) |  |  |
