# Supplementary material for: A multi-institutional study of bladder-preserving therapy for stage II-IV bladder cancer: A Korean Radiation Oncology Group Study (KROG 14-16)
Source: PLoS One. 2019 Jan 17;14(1):e0209998. doi: 10.1371/journal.pone.0209998 (PMC6336268; doi:10.1371/journal.pone.0209998)
Supplement: S4 Table — (DOCX) [file pone.0209998.s007.docx]

**S4 Table. Clinical outcomes according to clinical T stage.**

| **Variables** | **T2** | **T3/T4** | **p-value** |
| --- | --- | --- | --- |
| **Clinical N stage** |  |  |  |
| Negative | 72 (96.0%) | 53 (68.8%) | < 0.001 |
| Positive | 3 (4.0%) | 24 (31.2%) |  |
| **Gross residual tumor after TURBT** |  |  |  |
| Present | 41 (57.7%) | 58 (82.9%) | 0.001 |
| Absent | 30 (42.3%) | 12 (17.1%) |  |
| **Response after radiotherapy** |  |  |  |
| Complete response | 38 (54.3%) | 31 (41.9%) | 0.472 |
| Partial response | 19 (27.1%) | 27 (36.5%) |  |
| Progression | 6 (8.6%) | 9 (12.2%) |  |
| Stable disease | 7 (10.0%) | 7 (9.4%) |  |
| **Survival rates** |  |  |  |
| 5-year overall survival | 58.3% | 33.8% | 0.022 |
| 5-year cause-specific survival | 62.9% | 35.2% | 0.01 |
| 5-year disease-free survival | 28.1% | 14.0% | 0.078 |

Abbreviations: TURBT = transurethral resection of bladder tumor.
