# Supplementary material for: A multi-institutional study of bladder-preserving therapy for stage II-IV bladder cancer: A Korean Radiation Oncology Group Study (KROG 14-16)
Source: PLoS One. 2019 Jan 17;14(1):e0209998. doi: 10.1371/journal.pone.0209998 (PMC6336268; doi:10.1371/journal.pone.0209998)
Supplement: S5 Table — (DOCX) [file pone.0209998.s008.docx]

**S5 Table. Univariate analysis of prognostic factors to OS, CSS and DFS.**

| **Variables** | **No.** | **5Y OS** | **p-value** | **5Y CSS** | **p-value** | **5Y DFS** | **p-value** |
| --- | --- | --- | --- | --- | --- | --- | --- |
| **Age** |  |  |  |  |  |  |  |
| ≤ 72 | 80 | 52.2% | 0.006 | 53.6% | 0.023 | 26.4% | 0.036 |
| > 72 | 72 | 38.4% |  | 43.4% |  | 14.5% |  |
| **Initial hemoglobin** |  |  |  |  |  |  |  |
| ≤ 12 g/dL | 75 | 32.5% | 0.005 | 37.1% | 0.011 | 17.5% | NS |
| > 12 g/dL | 77 | 58.4% |  | 59.5% |  | 24.0% |  |
| **Tumor multiplicity** |  |  |  |  |  |  |  |
| Solitary | 97 | 49.8% | 0.078 | 52.0% | NS | 22.1% | NS |
| Multiple | 46 | 33.4% |  | 38.3% |  | 14.9% |  |
| **Clinical T stage** |  |  |  |  |  |  |  |
| 2 | 75 | 58.3% | 0.01 | 62.9% | 0.005 | 28.1% | NS |
| 3 | 54 | 39.0% |  | 40.5% |  | 15.5% |  |
| 4 | 23 | 21.7% |  | 22.9% |  | 10.4% |  |
| **Clinical N stage** |  |  |  |  |  |  |  |
| Negative | 125 | 48.4% | 0.032 | 52.2% | 0.008 | 23.8% | 0.017 |
| Positive | 27 | 33.0% |  | 33.0% |  | 7.4% |  |
| **Clinical stage group** |  |  |  |  |  |  |  |
| Ⅱ | 72 | 56.8% | 0.014 | 61.4% | 0.002 | 27.7% | 0.02 |
| Ⅲ | 49 | 40.7% |  | 43.4% |  | 20.5% |  |
| Ⅳ | 31 | 27.6% |  | 27.6% |  | 6.5% |  |
| **Hydronephrosis** |  |  |  |  |  |  |  |
| Present | 40 | 27.5% | 0.005 | 28.2% | 0.003 | 12.0% | NS |
| Absent | 111 | 52.2% |  | 56.4% |  | 24.5% |  |
| **Tumor response to RT** | |  |  |  |  |  |  |
| CR | 69 | 58.9% | < 0.001 | 64.6% | < 0.001 | 45.9% | < 0.001 |
| Non-CR | 75 | 35.0% |  | 35.9% |  | 0% |  |
| **Concurrent chemotherapy** | |  |  |  |  |  |  |
| Yes | 97 | 50.8% | NS | 53.7% | NS | 26.1% | 0.024 |
| No | 55 | 35.4% |  | 39.1% |  | 11.2% |  |

Abbreviations: OS = overall survival; CSS = cause-specific survival; DFS = disease-free survival; Y = year; RT = radiotherapy; CR = complete response.
