# Supplementary material for: A multi-institutional study of bladder-preserving therapy for stage II-IV bladder cancer: A Korean Radiation Oncology Group Study (KROG 14-16)
Source: PLoS One. 2019 Jan 17;14(1):e0209998. doi: 10.1371/journal.pone.0209998 (PMC6336268; doi:10.1371/journal.pone.0209998)
Supplement: S6 Table — (DOCX) [file pone.0209998.s009.docx]

**S6 Table. Multivariate analysis of prognostic factors of OS, CSS, and DFS.**

|  |  | **OS** | | **CSS** | | **DFS** | |
| --- | --- | --- | --- | --- | --- | --- | --- |
|  | **No.** | **HR** | **95% CI** | **HR** | **95% CI** | **HR** | **95% CI** |
| **Age** |  |  |  |  |  |  |  |
| ≤ 72 | 80 | 0.51 | 0.32 - 0.80 | 0.56 | 0.35 – 0.90 | - | - |
| > 72 | 72 |  |  |  |  |  |  |
| **Tumor response to RT** |  |  |  |  |  |  |  |
| CR | 69 | 0.48 | 0.30 – 0.76 | 0.43 | 0.27 – 0.69 | - | - |
| Non-CR | 75 |  |  |  |  |  |  |
| **Concurrent chemotherapy** |  |  |  |  |  |  |  |
| Yes | 97 | - | - | - | - | 0.66 | 0.44 – 0.99 |
| No | 55 |  |  |  |  |  |  |

Abbreviations: OS = overall survival; CSS = cause-specific survival; DFS = disease-free survival; HR = hazard ratio; CI = confidence interval; CR = complete response.
